# Robust Internal Elastic Lamina Fenestration in Skeletal Muscle Arteries

**DOI:** 10.1371/journal.pone.0054849

**Published:** 2013-01-24

**Authors:** Brett S. Kirby, Allison Bruhl, Michelle N. Sullivan, Michael Francis, Frank A. Dinenno, Scott Earley

**Affiliations:** 1 Department of Biomedical Sciences, Vascular Physiology Research Group, Colorado State University, Fort Collins, Colorado, United States of America; 2 Department of Health and Exercise Science, Human Cardiovascular Physiology Laboratory, Colorado State University, Fort Collins, Colorado, United States of America; 3 Department of Physiology, University of South Alabama College of Medicine, Mobile, Alabama, United States of America; Idaho State University, United States of America

## Abstract

Holes within the internal elastic lamina (IEL) of blood vessels are sites of fenestration allowing for passage of diffusible vasoactive substances and interface of endothelial cell membrane projections with underlying vascular smooth muscle. Endothelial projections are sites of dynamic Ca^2+^ events leading to endothelium dependent hyperpolarization (EDH)-mediated relaxations and the activity of these events increase as vessel diameter decreases. We tested the hypothesis that IEL fenestration is greater in distal vs. proximal arteries in skeletal muscle, and is unlike other vascular beds (mesentery). We also determined ion channel protein composition within the endothelium of intramuscular and non-intramuscular skeletal muscle arteries. Popliteal arteries, subsequent gastrocnemius feed arteries, and first and second order intramuscular arterioles from rat hindlimb were isolated, cut longitudinally, fixed, and imaged using confocal microscopy. Quantitative analysis revealed a significantly larger total fenestration area in second and first order arterioles vs. feed and popliteal arteries (58% and 16% vs. 5% and 3%; N = 10 images/artery), due to a noticeably greater average size of holes (9.5 and 3.9 µm^2^ vs 1.5 and 1.9 µm^2^). Next, we investigated via immunolabeling procedures whether proteins involved in EDH often embedded in endothelial cell projections were disparate between arterial segments. Specific proteins involved in EDH, such as inositol trisphosphate receptors, small and intermediate conductance Ca^2+^-activated K^+^ channels, and the canonical (C) transient receptor potential (TRP) channel TRPC3 were present in both popliteal and first order intramuscular arterioles. However due to larger IEL fenestration in first order arterioles, a larger spanning area of EDH proteins is observed proximal to the smooth muscle cell plasma membrane. These observations highlight the robust area of fenestration within intramuscular arterioles and indicate that the anatomical architecture and endothelial cell hyperpolarizing apparatus for distinct vasodilatory signaling is potentially present.

## Introduction

Skeletal muscle constitutes the largest mass of tissue in the body and notably, has a capacity for blood flow that far exceeds any other vascular bed when tissue metabolism is elevated [1]–[4]. Robust local vasodilation within the skeletal muscle resistance vasculature is responsible for this hyperemic response and is so extensive that calculated vasodilatory capacity can outstrip the pumping capacity of the heart [4], [5], and compromise mean arterial blood pressure if not restrained by sympathetic constriction [6]. Accordingly, rapid and dynamic mechanisms must act in tandem to finely control skeletal muscle arteriolar tone so that both tissue perfusion and mean arterial pressure are optimized. Recent data indicates that such control may involve divergent vasoregulatory mechanisms throughout the vascular network within the active skeletal muscle. Herein, evidence indicates the persistence of vasodilation in distal arterioles, whereas larger feed arteries are more prone to sympathetic vasoconstriction [7], [8]. While the exact means by which this phenomenon occurs is unclear, a number of studies suggest that smaller distal blood vessels have significant reliance on endothelium-dependent hyperpolarizing (EDH) mechanisms of vasodilation and larger vessels more dependent on nitric oxide (NO) [9], [10]. However, whether skeletal muscle contains the requisite vascular anatomical architecture and vasoactive machinery to support such dynamic vascular regulation is unclear.

Collective observations of vascular anatomy indicate the presence of an elastic tissue layer that separates the vascular endothelium from neighboring vascular smooth muscle cells, suitably termed the internal elastic lamina (IEL). More interestingly, while the IEL has physical barrier properties [11], [12], distinct fenestrae (or holes) are typically observed within this elastic sheath and may act as a “window” for enhanced communication between vascular endothelial and smooth muscle cell layers [13]. These areas of fenestration allow for passage of diffusible vasoactive substances and in some cases, the direct interface of endothelial cell membrane projections with underlying vascular smooth muscle [14]–[17]. In context to the latter, recent observations in mesenteric arteries indicate that some endothelial cell projections are sites of dynamic, spatially restricted Ca^2+^ events leading to EDH and subsequent relaxation [16]. Housed within projections are inositol trisphosphate receptors (IP_3_R) located on the endoplasmic reticulum (ER) and endothelial cell plasma membrane calcium-activated potassium channels (small and intermediate, KCa2.3 and KCa3.1; respectively), which provide the mechanism for evoking endothelial cell plasma membrane hyperpolarization. Also enriched at myoendothelial microdomains are transient receptor potential canonical 3 channels (TRPC3), which can aid in elevating endothelial intracellular Ca^2+^ levels [18]. Keeping in mind the function of skeletal muscle resistance arterioles compared to larger conduit arteries, we postulate that the presence of a highly fenestrated IEL coupled with an entrenched region for cellular hyperpolarization could allow for almost instantaneous and effortless communication of a vasomotor response. Whether such a schema exists in skeletal muscle has yet to be investigated.

Given that interventions to acutely elevate blood flow and/or shear stress stimuli induce an in increase in IEL fenestration [11], [17], [19], and the natural exposure of skeletal muscle vasculature to such stress, we tested the hypothesis that fenestration within the IEL is greater in distal arterioles (intramuscular) vs. proximal arteries within skeletal muscle, and to a lesser extent in regions of more restricted dynamic changes in blood flow (mesentery). Further, we tested the hypothesis that the enhanced IEL fenestration in intramuscular arterioles are plentiful and embedded with proteins associated with EDH (KCa2.3, KCa3.1, IP_3_R, and TRPC3).

## Methods

### Animals

Male Sprague-Dawley rats (250–350 g; Harlan) were used for these studies. Animals were deeply anesthetized with pentobarbital sodium (50 mg ip) and euthanized by exsanguination according to a protocol approved by the Institutional Animal Care and Use Committees (IACUC) of Colorado State University. Hindlimbs were removed stored in ice cold 3-(N-morpholino) propanesulfonic acid (MOPS)-buffered saline (in mM): 3 MOPS (pH 7.4), 145 NaCl, 5 KCl, 1 MgSO_4_, 2.5 CaCl_2_, 1 KH_2_PO_4_, 0.02 EDTA, 2 pyruvate and 5 glucose and 1% bovine serum albumin. Popliteal arteries, feed arteries, and intramuscular 1^st^ and 2^nd^ arterioles (in the gastrocnemius) from the leg were dissected, cleaned of connective tissue and stored in MOPS buffered saline prior to further manipulation [20], [21]. In additional experiments, the mesentery was removed and 1^st^, 2^nd^, and 3^rd^ order arteries were dissected, cleaned, and similarly stored [22]. For clarification purposes, we refer to all vessels segments as arteries, yet when identifying differences between the skeletal muscle non intramuscular (popliteal and feed) and intramuscular (1^st^ and 2^nd^), we refer to the latter as arterioles. For IEL fenestration experiments, 5 animals where used wherein each specific artery was isolated from the right hindlimb or mesentery. Two regions of interest (ROI) per artery were analyzed equating to a total of 10 images for a given segment.

### Immunohistochemistry

Skeletal muscle and mesenteric arteries were cut lengthwise and mounted on Sylgard blocks with the endothelium exposed. The tissue was fixed with 4% formaldehyde for 20 minutes, permeabilized with 1% Triton-X 100, blocked with 2% bovine serum, albumin and incubated with primary antibodies of either anti-KCa3.1 [16], [23]–[26] (Alomone Labs #APC-064; Lot #AN-03; 1∶250), anti-KCa2.3 [23], [27]–[29] (Alomone Labs #APC-025; Lot #AN-04; 1∶250), anti-pan-IP3 Receptor [16], [30], [31] (Millipore #07-1210; Lot #NG1882874; 1∶250), or anti-TRPC3 [32], [33] (Abcam #ab63012; Lot #959308; 1∶250) overnight at 4°C. Arteries were probed with fluorescent secondary antibodies of either goat anti-rabbit conjugate Texas Red [34]–[36] (Abcam #ab6719, lot #GR34115-1; 1∶1000) for KCa3.1, KCa2.3, and IP3R or rabbit anti-sheep conjugate Texas Red [37], [38] (Abcam #ab6745, lot #GR29419-1; 1∶1000) TRPC3 for 1 hour at room temperature. As negative control experiments, artery segments were prepared as described above yet vehicle was added in place of the primary antibody. Secondary antibody was used as noted above. Immunofluorescence was detected using a Fluoview 1000 Olympus laser scanning confocal microscope equipped with spectral detectors; SIM Scanner, and optical zoom. A 60×/1.4 numerical aperture (NA) oil immersion objective was used for imaging. Autoflourescence of the IEL was excited using the 488-nm line of an argon laser, and emission was collected using the variable bandpass filter set at 500–530 nm. Texas Red fluorescent was detected using a 543-nm HeNe laser with variable bandpass filter set at 600–630 nm. DAPI fluorescent staining was imaged using a 405-nm laser controlled by the SIM scanner, and with the variable bandpass filter set at 475–575 nm. For 3-dimensional volume rendering, images were recorded in z-stacks at 1 µm increments from the base of the endothelium to the surface of the subintimal smooth muscle, and reconstructed using Volocity 6.0.0 (Perkin Elmer) [24], [39].

### Automated Analysis of Internal Elastic Lamina Fenestration

**Figure 1:**
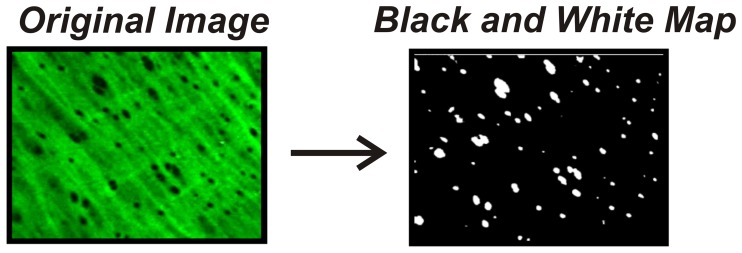
Depiction of internal elastic lamina fenestration and conversion for fenestrae quantification. Autofluorescence of the internal elastic lamina was imaged via laser scanning confocal microscopy. Following 3-dimensional volume rendering, images were recorded and reconstructed using Volocity 6.0.0 to produce a black and white map which allowed for detection of fenestrae via Image J software analysis.

The average number, average size, and total area of fenestration in mesenteric and skeletal muscles arteries were examined in selected regions of flat IEL free of folding and calculated using a custom-designed macro (available via email from the corresponding author). For analysis, extended focus images of combined z-stacks were adjusted by contrast to clearly define the edges of each hole and converted into black and white maps (holes were black and IEL was white) via the Image J threshold command [40]. The Image J particle analyzer command [40] was used to measure the area of each hole, with all holes on the edge of an image excluded from analysis. The particle analyzer circularity parameter was used to exclude the analysis of non-circular grooves in the IEL and confine area analysis to the approximately circular holes. This dimensionless parameter determines the minimum circularity between zero (non-circular) and 1 (perfectly circular) for particle analysis, and was set at 0.5 for all mesenteric, popliteal, and feed arteries. For first order arterioles and second order arterioles, this parameter was set to 0 since fenestration deep in the vasculature are not circular. The total contact area was expressed as the percent of each image that is black, representing holes or open spaces in the IEL (See Figure 1). The Image J particle analyzer command uses an algorithm to assign best-fit ellipses around a locus of pixels above a threshold value. The best-fit ellipses have an area equal to the area of the pixel locus, even if it is non-circular. This is clear from the algorithm code itself, but it can also easily be verified with any image sequence containing a known pixel locus area. As described by Francis et al., [41] LC Pro was used to measure many computer-generated pixel loci and found no discrepancy between measured and known values.

### Isolated Blood Vessel Experiments

Skeletal muscle and mesenteric arteries were harvested and transferred to a vessel chamber (Living Systems). The proximal end of the vessel was cannulated with a glass micropipette and secured with monofilament thread. Blood was gently rinsed from the lumen, and the distal end of the vessel was cannulated and secured. Arteries were pressurized to 20 mmHg with PSS (in mM: 119 NaCl, 4.7 KCl, 1.8 CaCl_2_, 1.2 MgSO_4_, 24 NaHCO_3_, 0.2 KH_2_PO_4_, 10.6 glucose, and 1.1 EDTA) and superfused (5 ml/min) with warmed (37°C) PSS aerated with a normoxic gas mixture (21% O2, 6% CO2, balance N_2_). Following a 15-min equilibration period, intraluminal pressure was slowly increased to 80 mmHg, and vessels were stretched to remove bends. Following an additional 15-min equilibration period, inner diameter was continuously monitored using video microscopy and edge-detection software (Ionoptix). Arteries pressurized to 80 mmHg were exposed to isotonic PSS containing 60 mM KCl to assess viability of the preparation. Lastly, arteries were superfused with Ca^2+^-free PSS (in mM: 119 NaCl, 4.7 KCl, 1.2 MgSO_4_, 24 NaHCO_3_, 0.2 KH_2_PO_4_, 10.6 glucose, 1.1 EDTA, 3 EGTA, and 0.01 diltiazem) to determine maximum (i.e. passive) diameter [24], [39].

### Calculations and Statistics

All data are presented as mean ± SEM. Values of N refer to number of arteries for isolated vessel experiments or number of images analyzed for automated analysis of fenestration parameters. One-way ANOVA was used to compare the average number and size of fenestrae and the total open space between first, second, and third order mesenteric arteries and popliteal arteries, feed arteries, and first and second order arterioles isolated from rat hindlimb. When a significant F value was observed, the Student-Newman-Keuls *post hoc* test was utilized to determine specificity of significance. A level of *P*≤0.05 was accepted as statistically significant for all experiments.

## Results

### IEL Fenestration within Skeletal Muscle and Mesenteric Arteries

**Figure 2:**
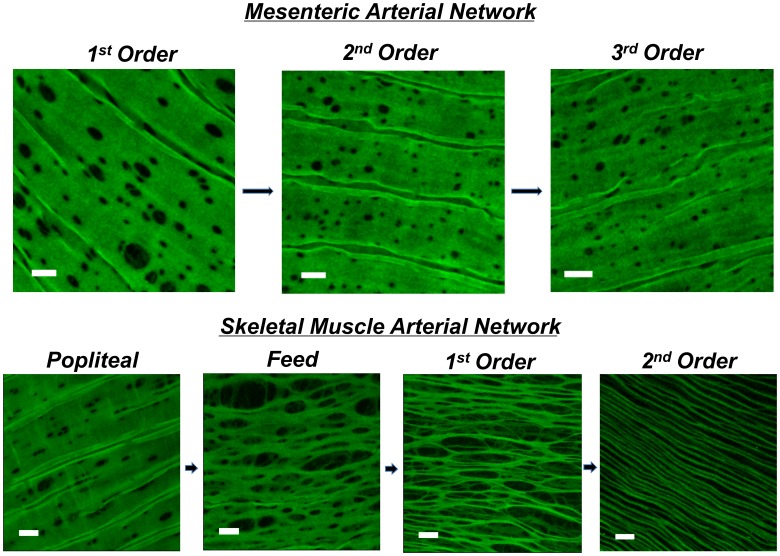
Internal elastic lamina in mesenteric vs. skeletal muscle arteries. Autofluorescence (green) of the internal elastic lamina (IEL) of mesenteric first, second, and third order arteries (top), and skeletal muscle popliteal, feed, first and second order arteries (bottom). Note that areas without green demonstrate lack of IEL, indicating fenestration and area of myoendothelial space. Bar = 10 µm.

**Figure 3:**
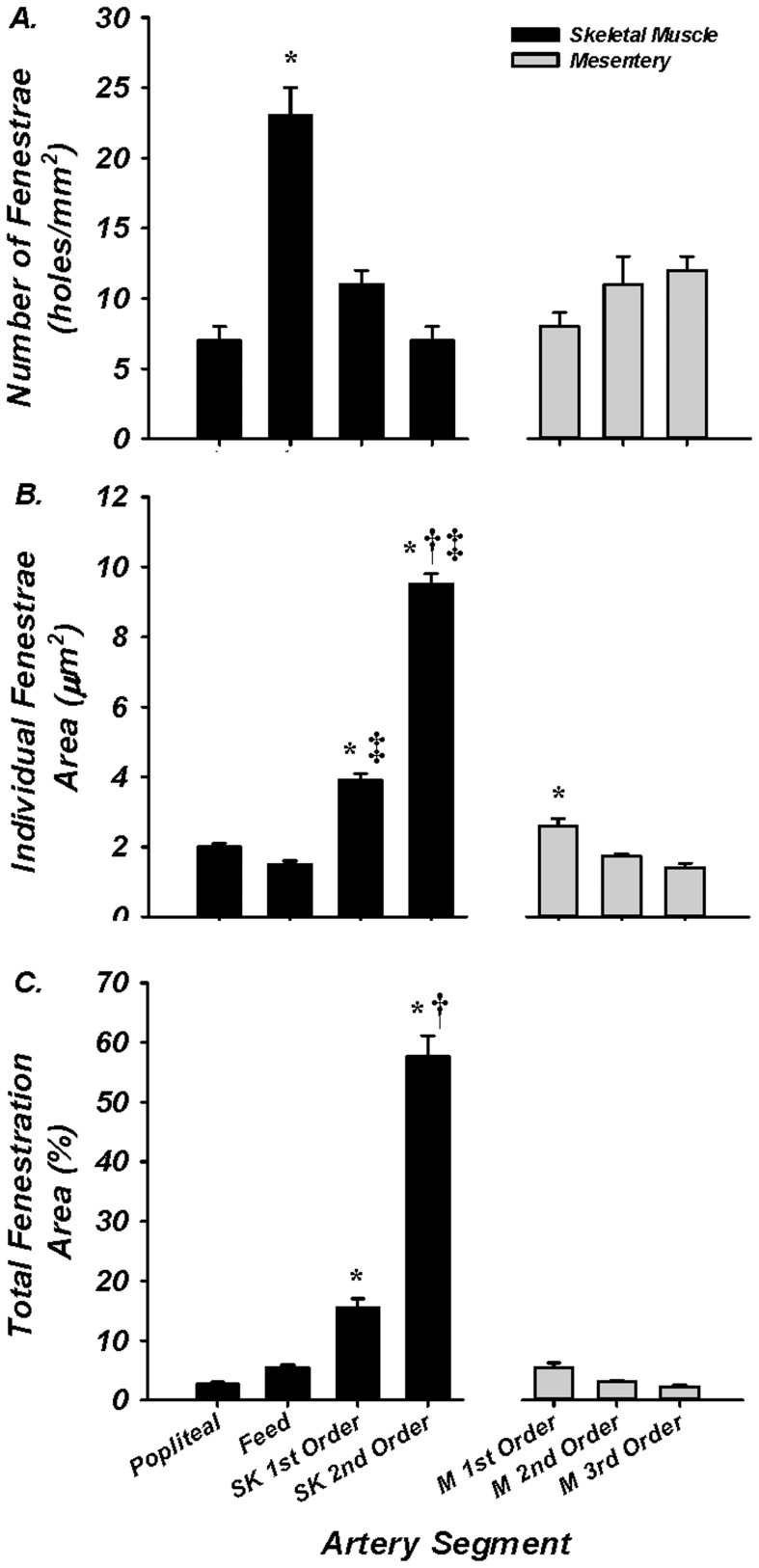
IEL fenestrae number, size, and total area. While all vessel segments had fenestrae, skeletal muscle feed arteries had a significantly greater number than all other vessel segments within skeletal muscle (black) and mesentery (gray); (A). Individual hole area for skeletal muscle first and second order arterioles and first order mesenteric arterioles was significantly different from all other vessel segments (B). Compilation of total fenestrae number and individual fenestrae size produced total MEC area as a percent. First and second order skeletal muscle arterioles had significantly greater area than all other artery segments. * = *P*<0.05 vs all other artery segments; ^†^ = *P*<0.05 vs 1^st^ order skeletal muscle arteriole; ^‡^ = *P*<0.05 vs 1^st^ order mesenteric artery.

The purpose of these experiments was to determine the number, size, and total area of IEL fenestrae in both proximal and distal segments of skeletal muscle and mesenteric arteries. Holes were present in all artery segments from each tissue bed (Figure 2). No significant difference in the number of holes was observed within the mesenteric network. In contrast, the skeletal muscle feed artery demonstrated a greater number of holes (23±2 holes/mm^2^; N = 10 images/artery) compared to the popliteal, 1^st^ order, 2^nd^ order skeletal muscle arterioles (7±1, 11±1, 7±1 holes/mm^2^, respectively; N = 10 images/artery) or 1^st^, 2^nd^, and 3^rd^ order mesentery (8±1, 11±2, 12±1 holes/mm^2^, respectively; N = 10 images/artery; Figure 3A). Distal intramuscular arterioles had significantly greater hole size (1^st^ and 2^nd^ order, 3.9±0.2 and 9.5±0.3 µm^2^; N = 10 images/artery) compared to its upstream vessel segments (popliteal = 2.0±0.1; feed = 1.5±0.1 µm^2^; 10 images/artery; Figure 3B). Within the mesentery, the average individual hole size was greatest in the 1^st^ order artery compared to 2^nd^ and 3^rd^ order arteries (2.6±0.2, 1.7±0.1, 1.4±0.1 µm^2^, respectively, N = 10 images/artery), and was greater than skeletal muscle popliteal and feed arteries, but smaller than the intramuscular 1^st^ and 2^nd^ order arterioles. Finally, as a compilation of hole number and size, total IEL fenestration area was calculated. All mesenteric arteries and the skeletal muscle popliteal and gastrocnemius feed arteries had less than 5% total IEL fenestration area (i.e. 95% of the lamina is non-fenestrated and continuous). In contrast, both intramuscular 1^st^ order (16±2%) and 2^nd^ order arterioles (58±4%) had significantly greater total fenestration area compared to their respective upstream vessel segments, as well as all arteries of the mesentery (Figure 3C). Furthermore, the skeletal muscle 2^nd^ order arteriole had significantly greater total fenestration area vs the 1^st^ order arteriole. These data support the hypothesis that total IEL fenestration area is robust in a vascular bed of tremendous vasodilatory capacity [1]–[4].

### Artery Diameter along Skeletal Muscle and Mesenteric Vascular Networks

**Figure 4:**
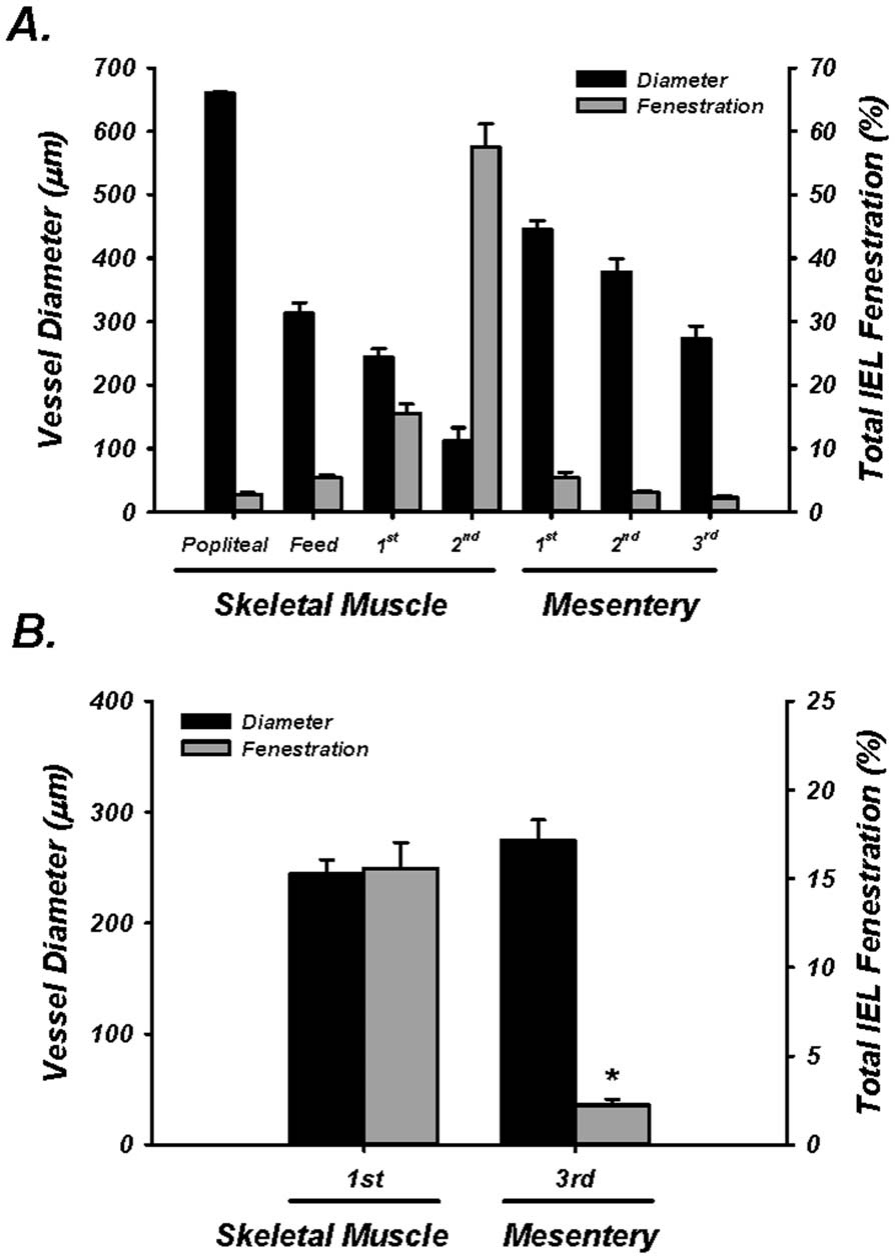
Vessel diameter vs. total IEL fenestration. A: Diameter (black bars) and total IEL Fenestration (gray bars) for skeletal muscle and mesenteric arteries for all vessels. B: Diameter (black bars) and total IEL Fenestration (gray bars) for size-matched 1^st^ order skeletal muscle arterioles and 3^rd^ order mesenteric arteries. * = *P*<0.05 vs skeletal muscle.

It has been suggested that the number of endothelial cell projections increase as vessel diameter decreases along the vascular network [42]. Therefore, we determined arterial diameter in multiple segments along the skeletal muscle and mesenteric vascular networks. The luminal diameter of skeletal muscle vessels pressurized to 80 mmHg was 661±1, 313±17, 244±13, and 112±21 µm for the popliteal, feed, 1^st^ order and 2^nd^ order skeletal muscle arterioles, respectively. All skeletal muscle vessels were significantly different from each other (*P*<0.05). The 1^st^, 2^nd^, and 3^rd^ order mesenteric arteries were also all different from each other (446±13, 378±21, and 274±19 µm, respectively; (*P*<0.05) (Figure 4A). More importantly, the mean diameter of the 3^rd^ order mesenteric artery was not significantly different from either the skeletal muscle feed or intramuscular 1^st^ order arterioles (*P* = 0.202 and 0.264) (Figure 4B). These findings clearly demonstrate that the absolute blood vessel diameter *per se* is not explanatory for the observed differences in total IEL fenestration area and structure within the skeletal muscle arterial bed.

### Presence of Proteins Associated with Endothelium-Dependent Hyperpolarization in Skeletal Muscle Arteries

**Figure 5:**
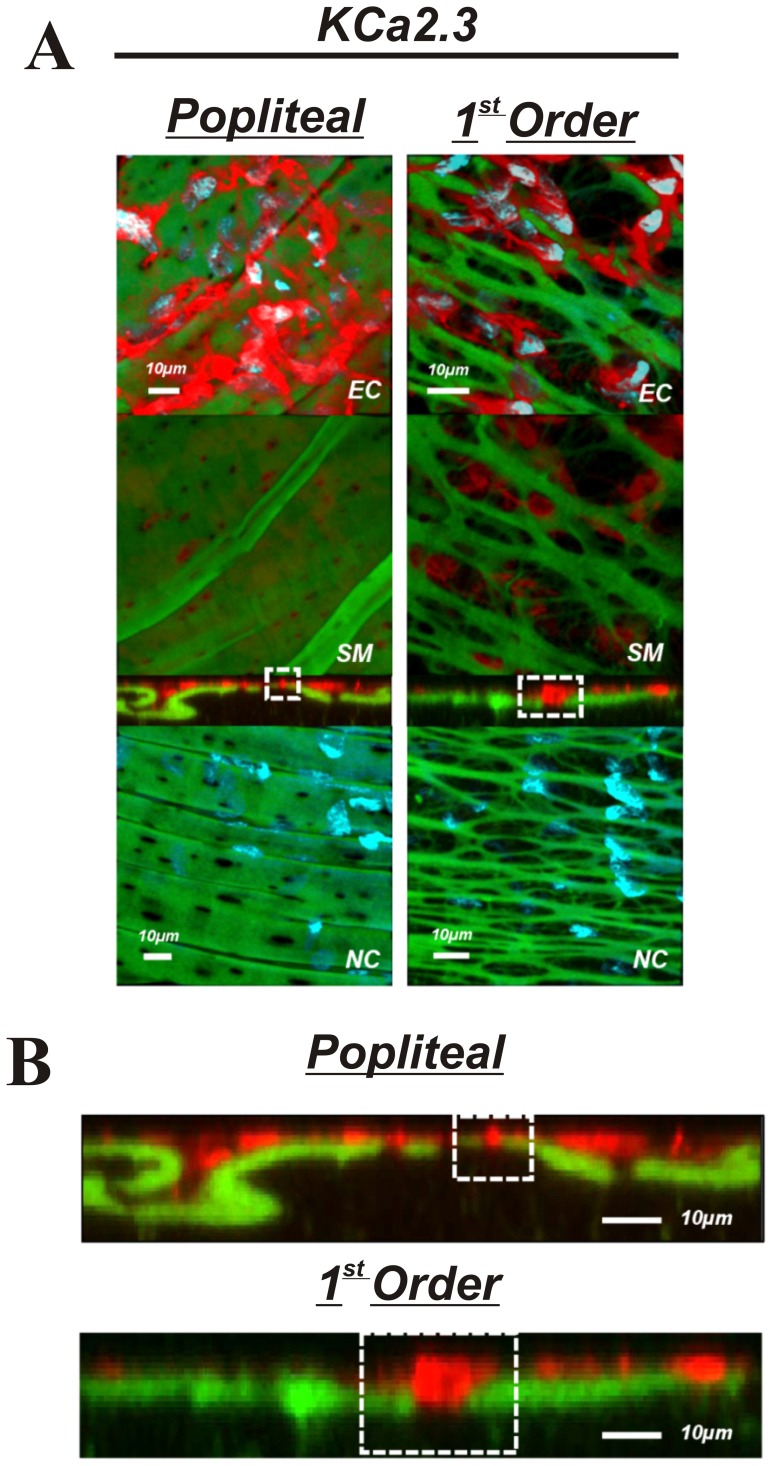
KCa2.3 in skeletal muscle arteries. A: Skeletal muscle popliteal (left) and 1^st^ order (right) arteries immunolabeled for KCa2.3 (top 3 panels) or no primary antibody control (bottom panel). The top image represents a composite of DAPI-stained nuclei in the endothelium, autofluorescence of the internal elastic lamina (green), and KCa2.3 immunolabeling (red), and a compressed z-stack image endothelial cell surface (EC, top panel). The second image is a compressed z-stack image from the perspective of the smooth muscle face (SM), the third image is a cross-sectional slice, and the fourth is a compressed z-stack image of a no primary antibody control (NC). Red staining indicates endothelial expression of KCa2.3. B: Magnified image of the cross-sectional view of the IEL. Note the sections highlighted by dotted square boxes indicating larger MEC and area of KCa2.3 staining in 1^st^ order arterioles. Bar = 10 µm. Representative of images from tissue isolated from at least 3 animals.

**Figure 6:**
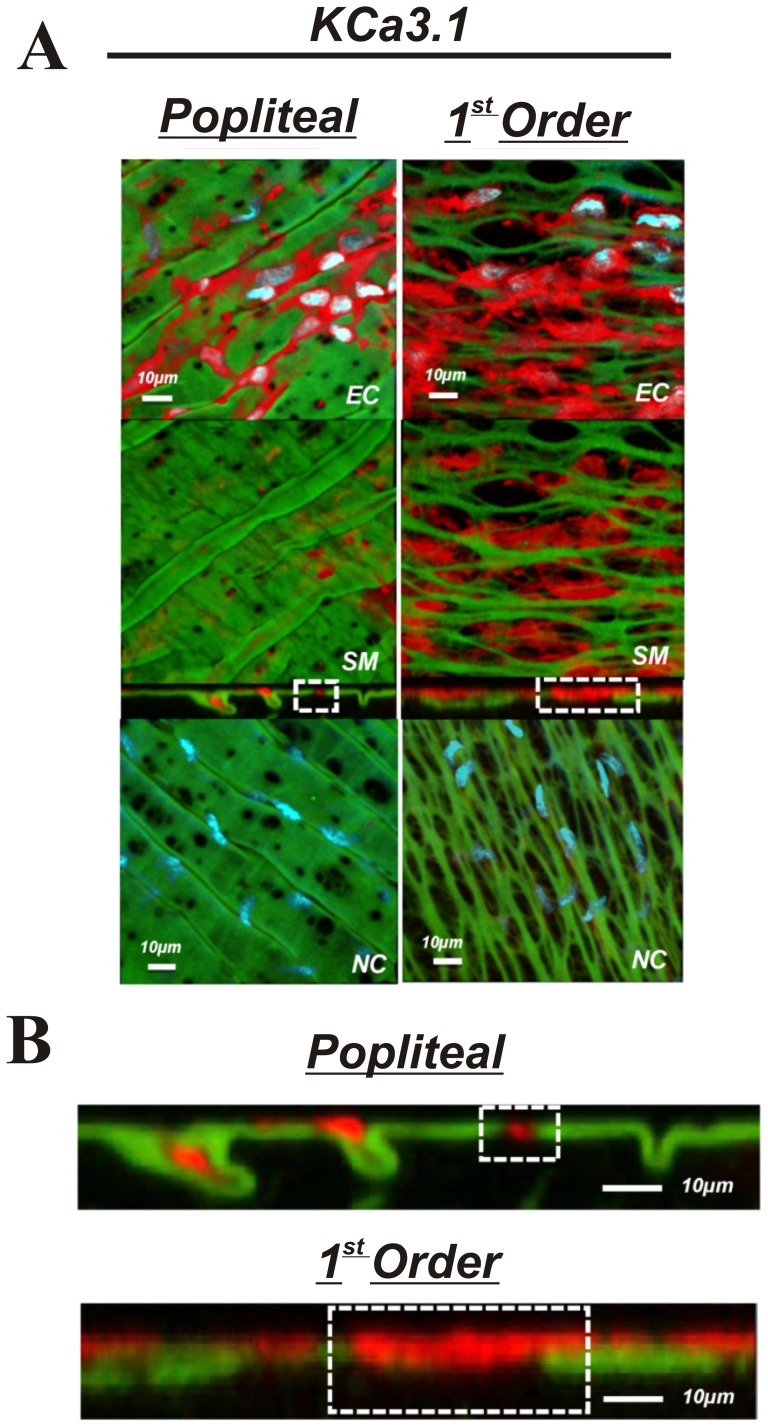
KCa3.1 in skeletal muscle arteries. A: skeletal muscle popliteal (left) and 1^st^ order (right) arteries immunolabeled for KCa3.1 (top 3 panels) or no primary antibody control (bottom panel). The top image represents a composite of DAPI-stained nuclei in the endothelium, autofluorescence of the internal elastic lamina (green), and KCa3.1 immunolabeling immunolabeling (red), and a compressed z-stack image endothelial cell surface (EC, top panel). The second image is a compressed z-stack image from the perspective of the smooth muscle face (SM), the third image is a cross-sectional slice, and the fourth is a compressed z-stack image of a no primary antibody control (NC).Red staining indicates endothelial expression of KCa3.1. B: Magnified image of the cross-sectional view of the IEL. Note the sections highlighted by dotted square boxes indicating larger MEC and area of KCa3.1 staining in 1^st^ order arterioles. Bar = 10 µm. Representative of images from tissue isolated from at least 3 animals.

**Figure 7:**
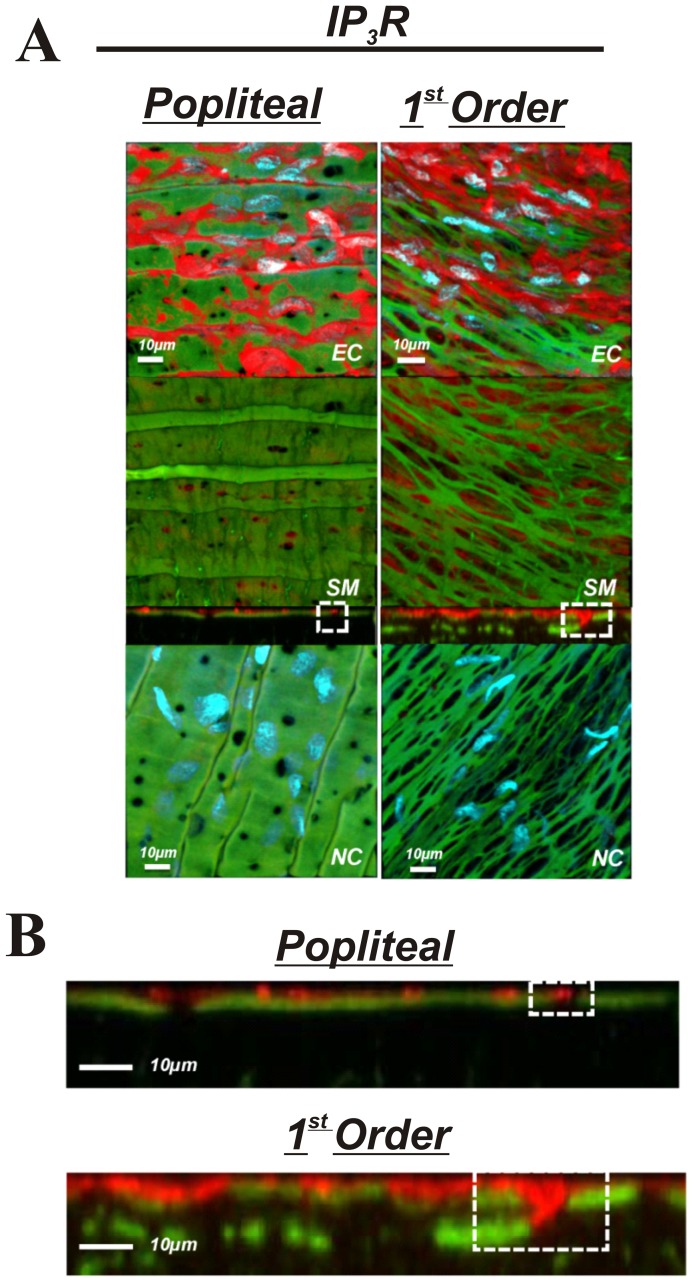
IP_3_R in skeletal muscle arteries. A: skeletal muscle popliteal (left) and 1^st^ order (right) arteries immunolabeled for IP_3_R (top 3 panels) or no primary antibody control (bottom panel). The top image represents a composite of DAPI-stained nuclei in the endothelium, autofluorescence of the internal elastic lamina (green), and IP_3_R immunolabeling immunolabeling (red), and a compressed z-stack image endothelial cell surface (EC, top panel). The second image is a compressed z-stack image from the perspective of the smooth muscle face (SM), the third image is a cross-sectional slice, and the fourth is a compressed z-stack image of a no primary antibody control (NC).Red staining indicates endothelial expression of IP_3_R. B: Magnified image of the cross-sectional view of the IEL. Note the sections highlighted by dotted square boxes indicating larger MEC and area of IP_3_R staining in 1^st^ order arterioles. Bar = 10 µm. Representative of images from tissue isolated from at least 3 animals.

**Figure 8:**
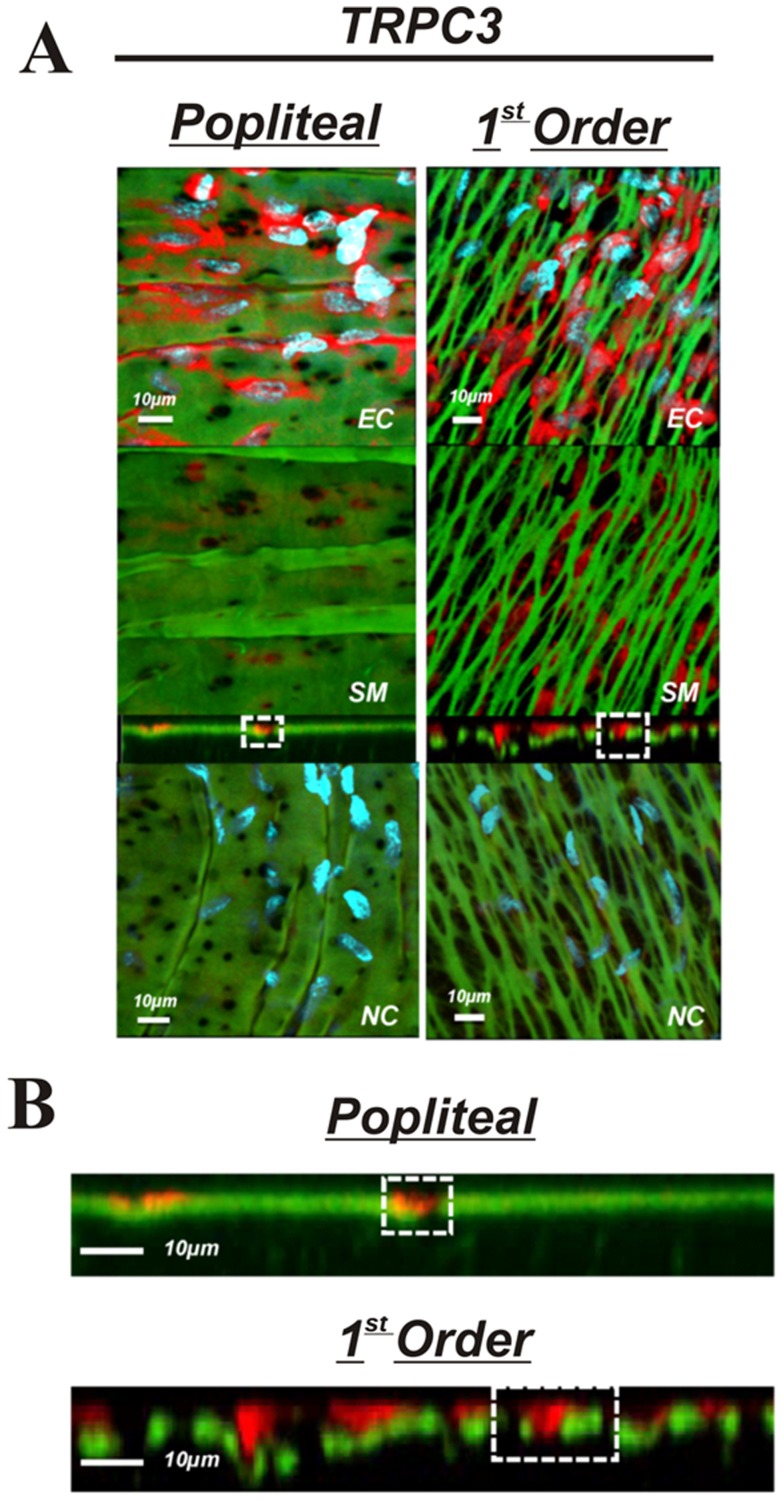
TRPC3 in skeletal muscle arteries. A: skeletal muscle popliteal (left) and 1^st^ order (right) arteries immunolabeled for TRPC3 (top 3 panels) or no primary antibody control (bottom panel). The top image represents a composite of DAPI-stained nuclei in the endothelium, autofluorescence of the internal elastic lamina (green), and TRPC3 immunolabeling immunolabeling (red), and a compressed z-stack image endothelial cell surface (EC, top panel). The second image is a compressed z-stack image from the perspective of the smooth muscle face (SM), the third image is a cross-sectional slice, and the fourth is a compressed z-stack image of a no primary antibody control (NC).Red staining indicates endothelial expression of TRPC3. B: Magnified image of the cross-sectional view of the IEL. Note the sections highlighted by dotted square boxes indicating larger MEC and area of TRPC3 staining in 1^st^ order arterioles. Bar = 10 µm. Representative of images from tissue isolated from at least 3 animals.

The purpose of these experiments was to determine if presence, localization, or abundance of specific proteins associated with EDH differs between large non-intramuscular and small intramuscular arterioles. Comparisons were made between popliteal and first order skeletal muscle arterioles. The presence K_Ca_2.3, K_Ca_3.1, IP_3_R, and TRPC3 was observed in both the popliteal and 1^st^ order arterioles (Figures 5,6,7 & 8) indicating the functional machinery for EDH exists mutually along the arterial network within skeletal muscle. Notably however, the *area* of fluorescent stain for all proteins appears greater in the 1^st^ order arteriole compared to the popliteal artery, coinciding with amount of IEL, likely indicating a greater spanning area of myoendothelial projection and therefore greater contact (Figures 5–8B). Similarly, it is important to observe that each protein (red label) penetrates deeper within the IEL (green) of the 1^st^ order arterioles, and is therefore presumably suggestive of larger myoendothelial projection size (Figures 5–8B).

## Discussion

The present investigation is the first to quantitatively characterize IEL fenestration along multiple segments of the skeletal muscle arterial network. Consistent with others [15], we observe modest IEL fenestration (≤5%) in multiple locations along the mesenteric vasculature. However, while the skeletal muscle conduit (popliteal) arteries reveal a somewhat similar IEL fenestration to mesenteric arteries, we show for the first time that skeletal muscle feed arteries have significantly large number of IEL holes, and that a vastly different and significantly greater total IEL fenestration area occurs within the intramuscular 1^st^ order and 2^nd^ order resistance arterioles (∼15% and ∼55%, respectively) compared to their respective upstream arteries or the mesenteric arterial network. Specifically, these smaller skeletal muscle resistance vessels have an apparent plexus or mesh-like IEL as opposed to distinct holes that are typically observed in other vascular beds. We demonstrate that this alteration in IEL fenestration does not correlate with vessel diameter *per se*, but appears to be a unique property of intramuscular resistance arterioles. To expand on these observations, we further aimed to determine whether proteins involved in EDH, such as, K_Ca_2.3, K_Ca_3.1, IP_3_R, and TRPC3, were also incongruent along the skeletal muscle arterial tree. The presence of these EDH proteins is similar in both large skeletal muscle conduit arteries (popliteal) as well as small first order arterioles, as evidenced by our immunostaining data in Figures 5–8. However, due to the significant lack of IEL in the resistance arterioles, a noticeably larger area of channel protein is observed (see smooth muscle face of Figures 5–8), and in theory, could offer less barrier to intercellular vasomotor communication. Additionally, an enhanced view of cross-sectional images suggests the possible presence of larger myoendothelial projections in the small intramuscular arterioles vs. the larger skeletal muscle conduit arteries. This raises the point that anatomy rather than simply EDH ‘machinery’ per se, may also have an impact on observed differences in vasodilatory signaling between small and large arteries. Importantly however, such a postulate has yet to be definitively tested. Collectively, these observations highlight the robust area of fenestration within intramuscular arterioles and indicate that the anatomical architecture and EDH apparatus for distinct vasodilatory signaling is potentially present within skeletal muscle of the rat hindlimb.

The rationale for the present investigation stems from the massive vasodilatory capacity within active skeletal muscle [4], [5], and from the understanding that rapid and precisely controlled regulation of blood flow is needed to match the ever changing metabolic demand for oxygen within this tissue [43], [44]. From this foundation, we reasoned that the vascular anatomical architecture associated with vasomotor tone [14] could be divergent between skeletal muscle and a bed of lower maximal blood flow capacity, such as the mesenteric circulation. In agreement with this underlying principle, we observe a considerably different pattern of IEL fenestration within skeletal muscle arterial network compared to that of the mesenteric, whereby along the vascular tree from the conduit artery to the 2^nd^ order arteriole, distinct holes transition into a more plexus like appearance. This was not observed in the mesenteric network, as small holes in the IEL were seen in all segments of this vascular bed. Similarly, small IEL holes are also detected in the cerebral vasculature [24]; a vascular bed recognized for relatively steady flow. These data are supported by evidence indicating that acute interventions aimed to elevate blood flow in non-resistance vessels (carotid) stimulate in increased IEL fenestration, while reducing blood flow accelerates a decrease in IEL fenestrae size [17]. Evidence suggests that high shear stress may stimulate changes in IEL fenestration by way of or repressing adhesion protein synthesis, lowering cell-matrix interactions, and thus consequently elevating IEL fenestration for means of increasing macromolecule exchange [19]. Regardless of the underlying mechanism, our data make clear that under normal (non-manipulated) physiological conditions the intramuscular resistance vasculature is replete with large IEL fenestrae.

IEL fenestration could in some circumstances act as an index for potential myoendothelial communication but it must be emphasized that the majority of data indicate that IEL holes sites and myoendothelial projections are not 1∶1 [14], [15], [42], [45]. Indeed, perusal of the cross-sectional images (Figures 5–8B) would support this concept as identification of penetrating protein within the IEL was not observed for every IEL hole-site. The IEL tissue layer parts the vascular endothelium from nearby vascular smooth muscle cells and in theory, less IEL could presumably allow for greater communication between cell layers resultant from the open IEL space (>50% Total IEL fenestration in the 2^nd^ order skeletal muscle arterioles; Figure 3C). In this respect, prior data demonstrate lack of lamina allows for greater molecule diffusion [12], thus aiding in the propensity for agents such as K^+^ ions, epoxyeicosatrienoic acids (EETs), nitric oxide or prostaglandins to act more readily between cell layers. In addition to diffusible myoendothelial crosstalk, direct physical interface through endothelial cell plasma membrane projections with vascular smooth muscle cells takes place through these fenestrae in the IEL [16], [42], [46], [47]. These projections house ER that spans throughout the length of the extension, of which is closely coupled as a microdomain with KCa2.3, KCa3.1, IP_3_R, and gap-junction forming connexin proteins, thus precisely placing the Ca^2+^ signaling and responsive apparatus in a location that would enable rapid cell-to-cell communication. Importantly, it is now clear that electrical events involving such an activation cascade are essential to initiate and sustain endothelial and vascular smooth muscle cell hyperpolarization and subsequent vasodilation [16], [23], [24].

Our observations confirm the presence of endothelial KCa2.3, KCa3.1, and IP_3_R located within the IEL fenestrae of skeletal muscle conduit and small resistance vessels and cross-sectional analysis indicates the likely presence of penetrating projections (Figures 4–8B). Furthermore, we extend these findings with the demonstration of endothelial TRPC3 embedded within the myoendothelial contact space (Figure 8), thought to be an important upstream signaling component participating in the increase in intracellular endothelial cell calcium levels necessary to activate KCa2.3 and KCa3.1 channels. Quantitative analysis of fluorescence intensity as an index of the amount of protein is problematic with immunostaining procedures. Nevertheless, as a result of less physical IEL barrier, a more obvious presence of endothelial cell protein is clearly observed (Figures 5–8; smooth muscle face), highlighting the potential importance of anatomical differences. Therefore, future study is warranted to determine whether our observations in skeletal muscle artery morphology are associated with EDH and divergent control of vessel tone.

We observed KCa2.3 channel expression in popliteal and skeletal muscle 1^st^ order arterioles that appears localized to the IEL fenestrae. Functional evidence suggests a crucial role for KCa2.3 in skeletal muscle hyperemia via hyperpolarizing mechanisms [48]. Our data corroborate these observations and demonstrate its localization to the myoendothelial space (Figure 6). While previous demonstration of KCa3.1 in these regions has been published [16], [24], KCa2.3 localization has often thought to predominate at the inter-endothelial junctions as evidenced by less discrete staining that outlines the endothelial cell body [23], [45]. Why these other studies did not observe KCa2.3 in the MEC space is not overly clear, however it should be emphasized that the present study is unique to skeletal muscle of the rat hindlimb; a region of muscle engagement during daily locomotion. Alternatively, antibody specificity could perhaps explain such observations. However, we did not observe staining in our negative control experiments indicating the selectivity of the compound (Figures 5–8A, bottom images). Additionally, we have previously shown no presence of KCa2.3 in cerebral arteries using the same antibody; hence we believe an authentic regional difference is involved. We speculate that KCa2.3 and KCa3.1 act in tandem to regulate the electrical activity of the endothelium within the skeletal muscle resistance vasculature [49].

### Considerations/Limitations

Previous investigation has hinted at greater myoendothelial cell projections as a function of decreasing vessel diameter within mesenteric arteries [42], [50]. Though IEL fenestrae and endothelial cell projections are not necessarily one in the same, we did not observe that vessel diameter *per se* was explanatory for our observations of greater IEL fenestrae, as descending vessel diameter did not occur concomitantly with greater fenestration in mesenteric arteries. Further, while 1^st^ order skeletal muscle arterioles and 3^rd^ order mesenteric arteries were of the same diameter, total fenestration area was significantly greater in the 1^st^ order skeletal muscle arterioles (Figure 4).

Protein content of each EDH protein assessed was observed in both skeletal muscle artery segments (popliteal and 1^st^ order). Moreover, cross-sectional viewing of this (Panels B of Figures 5–8) suggests that EDH protein is observed penetrating within the IEL of both vessel segments. In spite of this, given the greater total fenestration area of muscle arterioles (Figures 2 & 3), a larger projection could be present as evidenced by protein staining as an index (Figures 5–8). Further study is needed to substantiate such notions as our data do not provide ultimate evidence of such projections. These types of studies require finite experimentation and ultrastructural images obtained through the use of transmission electron microscopy, and are of interest to us for future study [51].

In the present study, we examined IEL fenestration in longitudinally opened and pinned arteries. It has been suggested by others that IEL properties should ideally be assessed under maximally dilated conditions to account for any differences in tone on the IEL [52], [53], thus our data is open to limitation. Nonetheless, all vessel segments studied in the present investigation were handled and examined in a consistent and uniform manner to allow for relative comparison.

Lastly, it should be noted that EDH-mediated dilation is observed in both mesenteric arteries [23], [54] as well as arteries from skeletal muscle [55]. This makes the case that anatomical differences between arterial beds cannot be a sole determinant underlying vasodilatory signaling.

### Perspective

**Figure 9:**
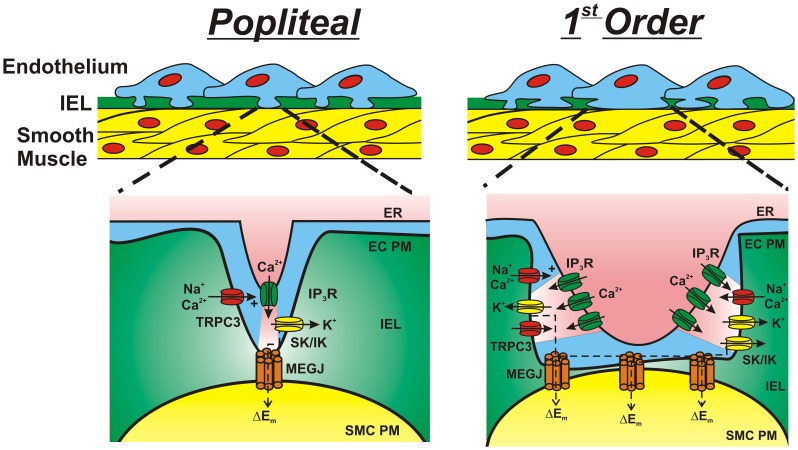
“Working hypothesis” schematic for electrical communication within large and small skeletal muscle arteries. Proposed vascular signaling among skeletal muscle popliteal (left) and 1^st^ order (right) arteries. ER, endoplasmic reticulum; EC PM, endothelial cell plasma membrane; IEL, internal elastic lamina; SMC PM, smooth muscle cell plasma membrane; IP3R, inositol trisphosphate receptor; SK, small conductance calcium-activated potassium channel (KCa2.3); IK, intermediate conductance calcium-activated potassium channel (KCa3.1); TRPC3, canonical transient receptor potential channel 3; MEGJ, myoendothelial gap junction.

Our morphological assessment and characterization of ion channel presence in skeletal muscle are important initial observations. Future understanding of the physiological and functional role these differences in IEL fenestrae poses equally critical. Our working hypothesis (Figure 9) is that the plexus like IEL found in intramuscular arterioles may provide less of a diffusional barrier to vasoregulatory molecules, and furthermore may allow a greater size or number [50] of endothelial cell projections may make direct contact with the vascular smooth muscle. Housed along the membrane of these endothelial cell protrusion lies TRPC3 channels that respond to G-coupled receptor stimulation and aid in raising intracellular Ca^2+^ through activation of IP3 receptors located on the ER. Following intracellular Ca^2+^ elevation, endothelial KCa channels are activated to hyperpolarize the plasma membrane and evoke a local and spreading vasodilation upstream from the intramuscular arterioles [54], [56]. We speculate that the large fenestration area in small resistance arterioles may in some manner aid in optimizing blood flow and oxygen delivery to skeletal muscle; a vascular bed that undergoes tremendous vasodilation during conditions such as exercise and therefore necessitating rapid and robust intra- and intercellular communication.

### Conclusions

The IEL fenestration within skeletal muscle arteries appears distinct from low-flow vascular beds such as the mesentery and is characterized by plexus like IEL (very high fenestration) in intramuscular arterioles. Given the presence of proteins involved in EDH, such as, KCa2.3, KCa3.1, IP_3_R, and TRPC3, throughout the entire skeletal muscle vascular network, and a greater area of exposure of these proteins due to less IEL barrier, these observations are suggestive that the anatomical architecture for distinct vasodilatory signaling is potentially present within skeletal muscle arteries.
